# Supplementary material for: MetaRibo-Seq measures translation in microbiomes
Source: Nat Commun. 2020 Jun 29;11:3268. doi: 10.1038/s41467-020-17081-z (PMC7324362; doi:10.1038/s41467-020-17081-z)
Supplement: Supplementary file 10 — Supplementary Data 7 [file 41467_2020_17081_MOESM10_ESM.zip › File2/Confidence_High_Taxonomy/20904_out.krona.html]

Javascript must be enabled to view this page.

members
magnitude
magnitudeUnassigned
count
unassigned
taxon
rank

20904\_out

3

2
3
superkingdom

phylum
3
1239

1

SRS104400\_contig\_number\_57826
1263002
species

186801
2
class

order
186802
2

family
2
31979

genus
420345
2

species

SRS062701\_contig\_number\_216SRS1055038\_contig\_number\_6572
341220
2
